# Evaluation of dose-volume histogram prediction for organ-at risk and planning target volume based on machine learning

**DOI:** 10.1038/s41598-021-82749-5

**Published:** 2021-02-04

**Authors:** Sheng xiu Jiao, Ming li Wang, Li xin Chen, Xiao-wei Liu

**Affiliations:** 1 School of Physics, Sun Yat-Sen University, 135 Xin Gang Road West, Guangzhou, 510275 China; 2 State Key Laboratory of Oncology in South China, Sun Yat-Sen University Cancer Center, 651 Dong Feng Road East, Guangzhou, 510060 China

**Keywords:** Cancer therapy, Radiotherapy

## Abstract

The purpose of this work is to evaluate the performance of applying patient dosimetric information induced by individual uniform-intensity radiation fields in organ-at risk (OAR) dose-volume histogram (DVH) prediction, and extend to DVH prediction of planning target volume (PTV). Ninety nasopharyngeal cancer intensity-modulated radiation therapy (IMRT) plans and 60 rectal cancer volumetric modulated arc therapy (VMAT) plans were employed in this study. Of these, 20 nasopharyngeal cancer cases and 15 rectal cancer cases were randomly selected as the testing data. The DVH prediction was performed using two methods. One method applied the individual dose-volume histograms (IDVHs) induced by a series of fields with uniform-intensity irradiation and the other method applied the distance-to-target histogram and the conformal-plan-dose-volume histogram (DTH + CPDVH). The determination coefficient R^2^ and mean absolute error (MAE) were used to evaluate DVH prediction accuracy. The PTV DVH prediction was performed using the IDVHs. The PTV dose coverage was evaluated using *D*_*98*_, *D*_*95*_, *D*_*1*_ and uniformity index (*UI*). The OAR dose was compared using the maximum dose, *V*_*30*_ and *V*_*40*_. The significance of the results was examined with the Wilcoxon signed rank test. For PTV DVH prediction using IDVHs, the clinical plan and IDVHs prediction method achieved mean *UI* values of 1.07 and 1.06 for nasopharyngeal cancer, and 1.04 and 1.05 for rectal cancer, respectively. No significant difference was found between the clinical plan results and predicted results using the IDVHs method in achieving PTV dose coverage (*D*_*98*,_ *D*_*95*,_ *D*_*1*_ and *UI*) for both nasopharyngeal cancer and rectal cancer (*p*-values ≥ 0.052). For OAR DVH prediction, no significant difference was found between the IDVHs and DTH + CPDVH methods for the R^2^, MAE, the maximum dose, *V*_*30*_ and *V*_*40*_ (*p*-values ≥ 0.087 for all OARs). This work evaluates the performance of dosimetric information of several individual fields with uniform-intensity radiation for DVH prediction, and extends its application to PTV DVH prediction. The results indicated that the IDVHs method is comparable to the DTH + CPDVH method in accurately predicting the OAR DVH. The IDVHs method quantified the input features of the PTV and showed reliable PTV DVH prediction, which is helpful for plan quality evaluation and plan generation.

## Introduction

With the continuous development of artificial intelligence and machine learning technology, a medical computerized clinical decision support and assistance systems based on more available clinical data have played an increasingly important role in helping clinicians make clinical decisions^1, 2^. In the field of radiotherapy, making dose-volume histogram (DVH) or dose distribution of organ at risk (OAR) predictions based on prior plan data could provide a valuable dose-volume reference that could help planners determine whether the quality of a treatment plan could be further improved^3–11^ and could be used as the dose-volume optimization input constraints in a treatment planning system (TPS) to assist in plan generation^12–17^. In addition, a machine learning method could predict the dose-volume parameter such as dose distribution index for treatment plan evaluation which is helpful for fast plan quality evaluation^18^.

The use of geometric information in predicting credible DVH has been widely studied; the representative patient geometric information descriptors are the overlap volume histogram (OVH) and the distance-to-target histogram (DTH). The OVH and DTH quantify the spatial relationship between OARs and the target^19–24^. Recently, an OAR DVH prediction method based on patient dosimetric information was proposed^25–27^, which indicated that using dosimetric information can improve DVH prediction.

In the treatment planning process, the dose-volume constraints of OAR and planning target volume (PTV) are needed for inverse optimization processes, and the PTV DVH prediction is beneficial for achieving clinically acceptable plans. By selecting a reference expansion target, Babier et al. used the OVH to predict the OAR and PTV DVH with the goal of automatically generating treatment plans for oropharynx patients^28^. The geometric information for the OAR, such as the DTH, was calculated based on the spatial relationship between the OAR and PTV and used to predict the OAR DVH. A few studies have reported PTV DVH prediction using DTH.

From another point of view, the individual DVHs of different fields containing the direction-dependent dosimetric information should be helpful for the DVH prediction. However, the effectiveness of the PTV DVH prediction and the OAR DVH prediction accuracy using the individual DVHs of different fields is unknown. This work is to evaluate the performance of using the individual DVHs of different fields in OAR DVH prediction, and to aim to give a method for PTV DVH prediction.

## Methods

In this work, the clinical treatment plans were used as the training and testing data. The different DVH prediction methods based on the geometric and dosimetric information were used to predict OAR DVH. The PTV DVH prediction was performed using only the dosimetric information. The prediction performance was evaluated using the dosimetric parameters, determination coefficient R^2^ and mean absolute error (MAE).

### Patient data

Following the Sun Yat-sen University Cancer Center Internal Review Board (IRB) approval (Approval No: YB2018-06), ninety90 nasopharyngeal carcinoma IMRT plans and 60 rectal cancer VMAT plans previously generated at our center were used as the database. Twenty nasopharyngeal carcinoma cases and 15 rectal cancer cases were randomly selected as the testing cases. The remaining cases were used as the training data. The informed consents have been obtained from all patients, and all patient data has been fully anonymized. All methods were performed in accordance with the relevant guidelines and regulations of the Sun Yat-sen University Cancer Center.

According to the guidelines of the Radiation Therapy Oncology Group (RTOG) protocols 0225 and 0615 for nasopharyngeal carcinoma and the RTOG protocol 0822 for rectal cancer, the dose-volume constraints for each structure were obtained and illustrated in Table 1. The nasopharyngeal carcinoma 9-field IMRT plans were generated with 6-MV photon beams using the Eclipse TPS (Varian Medical Systems, Palo Alto, USA, version 11.0). The gantry angles were: 160°, 120°, 80°, 40°, 0°, 200°, 240°, 280° and 320°. The target prescription dose of the nasopharyngeal carcinoma plan was 70 Gy in 32 fractions. Three PTVs, PTV70, PTV60 and PTV54 in the nasopharyngeal carcinoma IMRT plan were expanded by 3 mm from the corresponding clinical target volume (CTV70, CTV60 and CTV54). The OARs included the brainstem, spinal cord, chiasm, bilateral lens, bilateral optic nerve, bilateral parotid and bilateral temporal lobe. The rectal cancer plans were generated for double 6-MV VMAT arcs using the MONACO TPS (Elekta CMS, Maryland Heights, MO, version 5.10). The target prescription dose was 50 Gy in 25 fractions. Two PTVs, PTV50 and PTV45 in the rectal cancer VMAT plan were expanded by 5 mm from the CTV50 and CTV45, respectively. The OARs included the bladder, colon, bilateral femoral head and small intestine.

**Table 1:** The required dose optimization constraints for generating a radiation therapy plan.

Nasopharyngeal carcinoma IMRT plan		Rectal cancer VMAT plan	
Structure	Constraint	Structure	Constraint
PTV70	*D*_*0*_ ≤ 75 Gy	PTV50	*D*_*0*_ ≤ 55 Gy
–	*D*_*100*_ ≥ 71 Gy	–	*D*_*97*_ ≥ 51 Gy
–	*D*_*97*_ ≥ 72 Gy	–	*–*
PTV60	*D*_*100*_ ≥ 60 Gy	PTV45	*D*_*100*_ ≥ 45 Gy
PTV54	*D*_*100*_ ≥ 54 Gy	–	*–*
Brainstem	*D*_*0*_ ≤ 60 Gy	Bladder	*V*_*50*_ ≤ 50%
Spinal cord	*D*_*0*_ ≤ 50 Gy	–	*V*_*60*_ ≤ 20%
Chiasm	*D*_*0*_ ≤ 60 Gy	Colon	*D*_*0*_ ≤ 50 Gy
Lens	*D*_*0*_ ≤ 10 Gy	–	–
Optic nerve	*D*_*0*_ ≤ 60 Gy	Small intestine	*D*_*0*_ ≤ 50 Gy
Parotid	*V*_*30*_ ≤ 50%	–	*V*_*50*_ ≤ 5%
Temporal lobe	*D*_*0*_ ≤ 72 Gy	Femoral head	*V*_*50*_ ≤ 5%

*V*_*x*_ volume receiving greater than *x* Gy, *D*_*y*_ the dose to the highest *y*% of volume.

### DVH prediction method

The dosimetric information from individual fields with uniform-intensity radiation, termed the individual dose-volume histograms (IDVHs), was used to predict the OAR DVH and PTV DVH. Another method applied the DTH and the conformal plan dose-volume histogram (CPDVH) to predict the OAR DVH, which is referred to as the DTH + CPDVH method^26^. Table 2 shows the input features of the OAR and PTV used in this work.

**Table 2:** The geometric and dosimetric features of the OAR and PTV used in this study.

Structures	Features	Description
OAR	DTH CPDVH IDVHs	Distance-to-target histogram Conformal plan dose-volume histogram Individual dose-volume histograms
PTV	IDVHs	Individual dose-volume histograms

Each OAR has three features (DTH, CPDVH and IDVHs). The PTV has a single dosimetric feature (IDVHs).

### IDVHs method

The input of the IDVHs method was the IDVHs, which represents the individual dose-volume histograms of 9 fields without interfields dose superposition. For the nasopharyngeal carcinoma cases, the process of calculating the individual field dose was as follows: each field was fitted to the PTV54 and the dose was calculated using 6-MV photon beams in an Eclipse TPS. Nine equally spaced fields were used in the dose calculation. The slice thickness of the CTs was 0.3 cm. For VMAT technology with many fields, calculating the dose of a large number of individual fields is time consuming. To solve this problem, this work applied 9 equally spaced fields to calculate the dosimetric information in the rectal cancer cases. The individual field dose was calculated for 6-MV photon beams using the MONACO TPS. Each field was fitted to the PTV45. The slice thickness of the CTs was 0.3 cm. Each field had the same weight.

**Figure 1:**
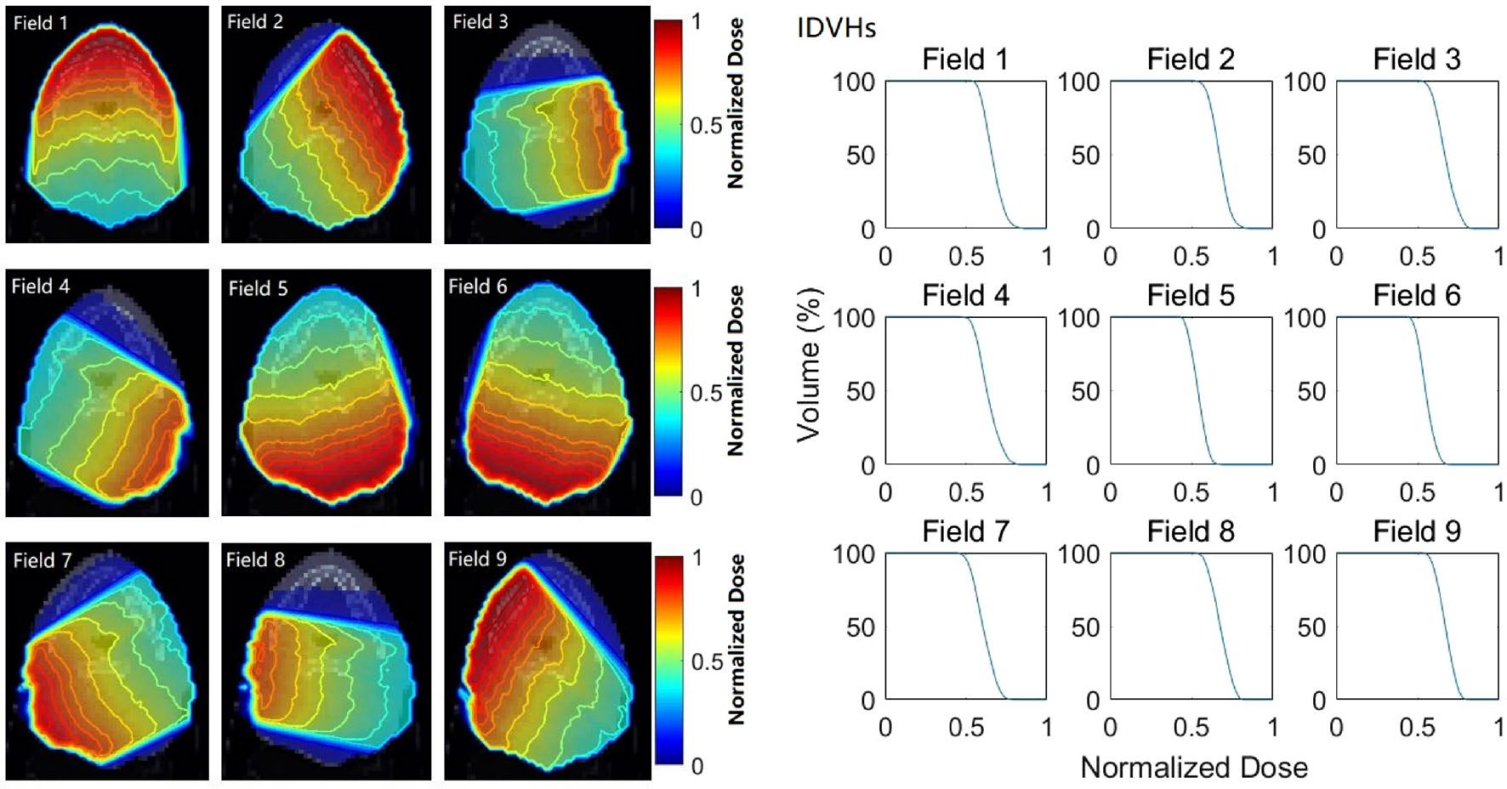
The dose distributions of the 9 individual fields with uniform-intensity irradiation at the same CT slice of a nasopharyngeal carcinoma patient (left) and the IDVHs of a highlighted structure (right).

Figure 1 illustrates the dose distributions of the 9 individual fields with uniform-intensity irradiation at the same CT slice of a nasopharyngeal carcinoma patient and the corresponding IDVHs of a highlighted structure. The IDVH dose was normalized to the maximum dose of all individual fields. As shown in Fig. 1, the dose fall-off rate information of each individual field could be clearly presented, which differs from previous reports on the small dose fall-off rate of the PTV boundary regions after interfield dose superposition^26^.

For the IDVHs method, the input consisted of 9 parts. Each part sampled 50 points from the cumulative DVH of each field at an equal-interval dose. Thus, the input of the IDVHs method was 450 dimensional. The DVH prediction model was a generalized regression neural network (GRNN)^29^, which was constructed using a neural network toolbox *nntool* of MATLAB (version R2018b, Math Works, Natick, MA). Of note, that the OAR and PTV were trained separately because the large difference of the DVH distribution of the OAR and PTV, and the multimodels may better predict the DVH^30^.

### DTH + CPDVH method

The DTH is used as the descriptor to quantify the spatial relationship between the OAR and PTV and represents the fractional volume of the OAR within a certain distance from the PTV surface^21^. To obtain the dosimetric information of each nasopharyngeal carcinoma patient, a 9-fields conformal plan was established. The process was as follows: the conformal plans were generated for 9 equally spaced fields with 6-MV photon beams. Each field was fitted to the PTV54 and had the same weight. All nasopharyngeal carcinoma conformal plans were developed using an Eclipse TPS. For the rectal cancer patients, the conformal plans were generated for 9 equally spaced fields with 6-MV photon beams. Each field was fitted to the PTV45 and had the same weight. All the rectal cancer conformal plans were developed using the MONACO TPS.

**Figure 2:**
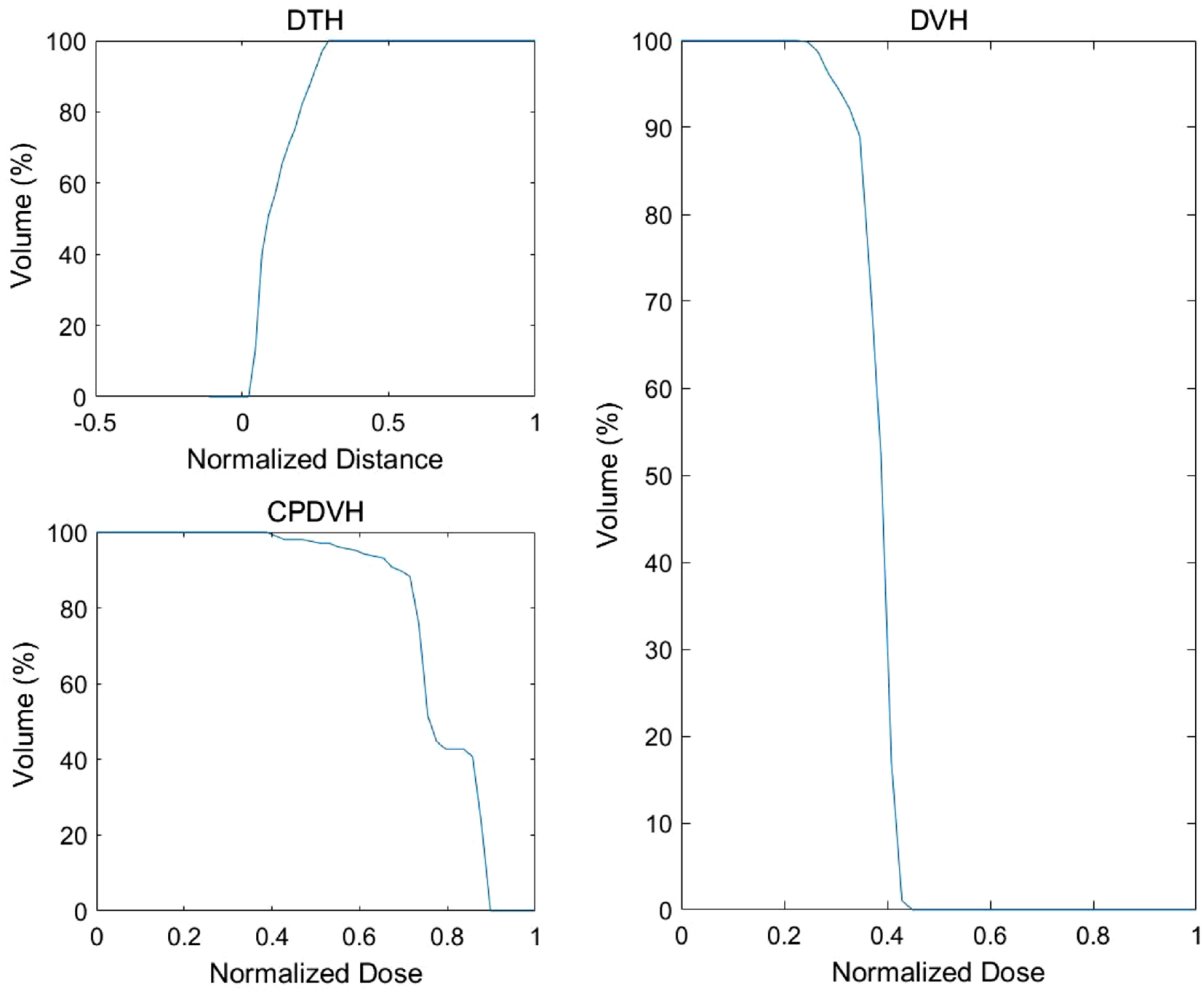
The DTH, CPDVH and DVH of an OAR.

**Figure 3:**
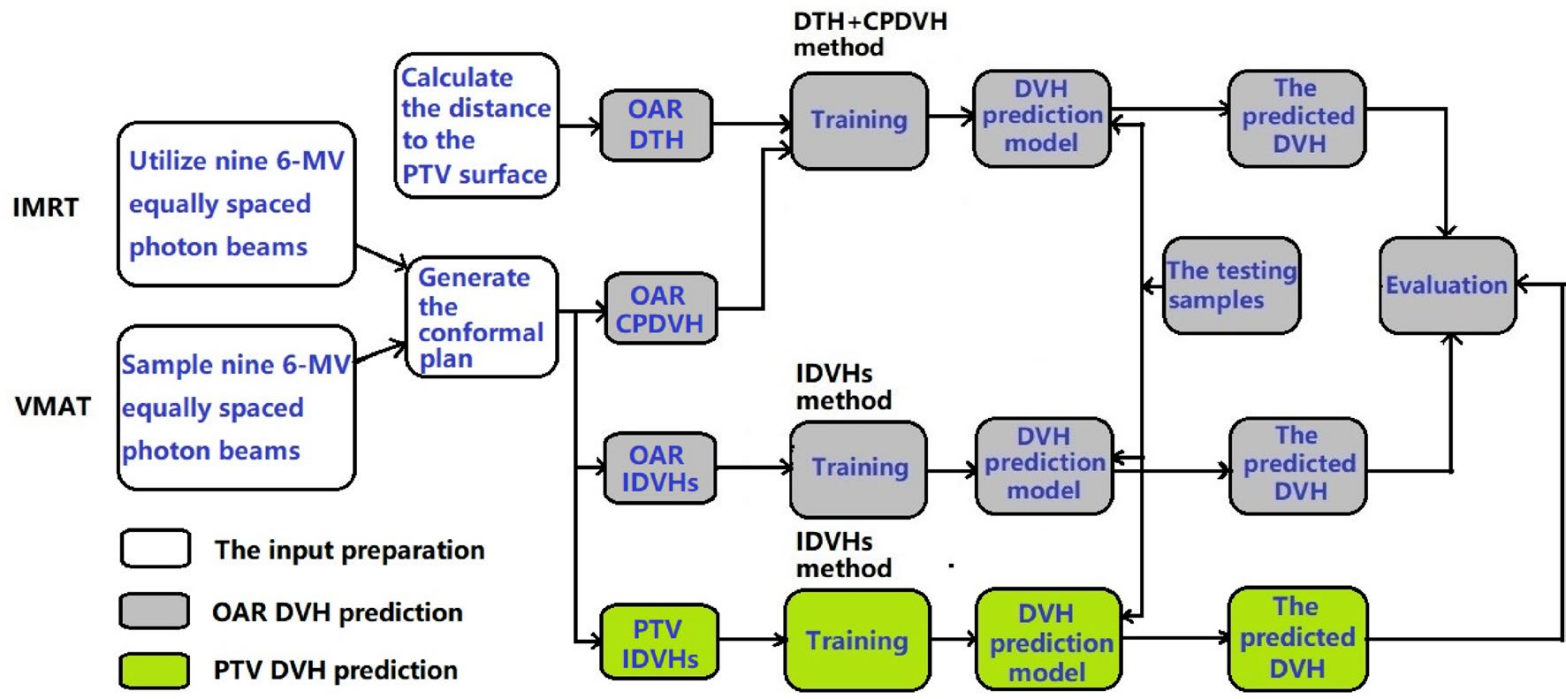
Flowchart showing the DVH prediction process.

In Fig. 2, the DTH distance was normalized to the maximum distance of all OARs. The CPDVH dose was normalized to the PTV maximum dose of all the conformal plans. The DVH dose was normalized to the PTV maximum dose of all the IMRT or VMAT plans. The input of the DTH + CPDVH method was 100 dimensional, which included 50 points from the cumulative DTH at equal-interval distance and 50 points from cumulative CPDVH at equal-interval dose. The DVH prediction model was GRNN. Figure 3 shows a flowchart of the OAR and PTV DVH prediction process.

### DVH prediction error

The DVH prediction accuracy of the DTH + CPDVH method and the IDVHs method was evaluated using the determination coefficient R^2^ and MAE. The closer R^2^ is to 1.0, and the closer MAE is to 0, the closer the predicted value is to the actual value. The parameter *D*_*98*_, *D*_*95*_, *D*_*1*_ and uniformity index (*UI*) were used to evaluate the PTV dose coverage. *D*_*y*_ is the dose to the highest *y*% of the volume. The OAR dosimetric result of the DTH + CPDVH prediction method and the IDVHs prediction method were evaluated using the maximum dose, *V*_*30*_ and *V*_*40*_. *V*_*x*_ represents the volume receiving greater than *x* Gy.

$$R^2 = {1 - }\frac{{\sum\limits_{i = {1}}^{{50}} {{{({V_{i,{\kern 1pt} TPS}} - {V_{i,{\kern 1pt} pred}})}^2}} }}{{\sum\limits_{i = {1}}^{{50}} {{{({V_{i,{\kern 1pt} TPS}} - \overline {{V_{{\kern 1pt} TPS}}} )}^2}} }}{\kern 1pt} {\kern 1pt} {\kern 1pt} {\kern 1pt} {\kern 1pt} {\kern 1pt} {\kern 1pt} {\kern 1pt} {\kern 1pt} {\kern 1pt} {\kern 1pt} {\kern 1pt} {\kern 1pt} {\kern 1pt} {\kern 1pt} {\kern 1pt} {\kern 1pt} {\kern 1pt} {\kern 1pt} {\kern 1pt} {\kern 1pt} {\kern 1pt} {\kern 1pt} {\kern 1pt} {\kern 1pt} {\kern 1pt} {\kern 1pt} {\kern 1pt} {\kern 1pt} {\kern 1pt} {\kern 1pt} {\kern 1pt} {\kern 1pt} {\kern 1pt} {\kern 1pt} {\kern 1pt}$$

$$MAE = \frac{{1}}{{{50}}}\sum\limits_{i = {1}}^{{50}} {\left| {{V_{i,{\kern 1pt} TPS}} - {V_{i,{\kern 1pt} pred}}} \right|} {\kern 1pt} {\kern 1pt} {\kern 1pt} {\kern 1pt} {\kern 1pt} {\kern 1pt} {\kern 1pt} {\kern 1pt} {\kern 1pt} {\kern 1pt} {\kern 1pt} {\kern 1pt} {\kern 1pt} {\kern 1pt} {\kern 1pt} {\kern 1pt} {\kern 1pt} {\kern 1pt} {\kern 1pt} {\kern 1pt} {\kern 1pt} {\kern 1pt} {\kern 1pt} {\kern 1pt} {\kern 1pt} {\kern 1pt} {\kern 1pt} {\kern 1pt} {\kern 1pt} {\kern 1pt} {\kern 1pt} {\kern 1pt} {\kern 1pt}$$

$$UI = \frac{{D_5}}{{D_{95}}}$$

${V_{i,TPS}}$ is the *i*th volume value in the DVH curve that was achieved by the TPS and ${V_{i,pred}}$ is the *i*th volume value in the DVH curve that was predicted by the DTH + CPDVH or IDVHs method. The *UI* values closer to 1 indicate better homogeneity^31^. Significant differences were tested using SPSS (version 17, IBM-SPSS Statistics, Inc., Chicago, IL). The Wilcoxon signed rank test was utilized to compare the difference. A *p*-value < 0.05 was considered statistically significant.

## Results

### OAR DVH prediction accuracy of the IDVHs method

The means and standard deviations of the R^2^ and MAE values for all testing cases are illustrated in Table 3. For nasopharyngeal cancer, the IDVHs method had a mean R^2^ ranging from 0.87 to 0.97 at all OARs with standard deviations ≤ 0.20. The IDVHs method had a mean R^2^ ≥ 0.92 for 5 out of 7 OARs in the 20 nasopharyngeal cancer test cases. The IDVHs method achieved a mean MAE value in the range from 1.16 to 7.95% with standard deviations ≤ 6% at all OARs. The IDVHs method produced a mean MAE ≤ 4.5% for 5 out of 7 OARs in the 20 nasopharyngeal cancer test cases. No significant differences in the R^2^ and MAE values between the DTH + CPDVH method and IDVHs method were found for the OARs in the 20 nasopharyngeal cancer test cases (p-value ≥ 0.218).

**Table 3:** The means and standard deviations of R^2^ and MAE of the DTH + CPDVH method and IDVHs method for 20 nasopharyngeal cancer patients and 15 rectal cancer patients.

OAR	R^2^			MAE (%)		
	DTH + CPDVH	IDVHs	p-value	DTH + CPDVH	IDVHs	p-value
**Nasopharyngeal cancer**						
Brainstem	0.97 ± 0.03	0.97 ± 0.03	0.647	3.52 ± 1.99	3.63 ± 2.24	0.650
Spinal cord	0.97 ± 0.03	0.97 ± 0.03	0.616	3.20 ± 1.50	3.22 ± 1.48	0.906
Chiasm	0.88 ± 0.24	0.87 ± 0.13	0.438	7.12 ± 8.00	7.95 ± 5.37	0.218
Lens	0.92 ± 0.08	0.92 ± 0.09	0.586	1.11 ± 0.88	1.16 ± 1.10	0.632
Optic nerve	0.89 ± 0.18	0.88 ± 0.16	0.679	6.84 ± 4.16	7.05 ± 4.82	0.831
Parotid	0.97 ± 0.03	0.97 ± 0.02	0.433	4.98 ± 3.12	4.34 ± 1.71	0.332
Temporal lobe	0.97 ± 0.07	0.97 ± 0.03	0.758	2.38 ± 1.95	2.41 ± 2.22	0.970
**Rectal cancer**						
Bladder	0.98 ± 0.03	0.97 ± 0.03	0.190	2.20 ± 1.42	2.36 ± 1.70	0.221
Colon	0.95 ± 0.04	0.95 ± 0.04	0.722	6.13 ± 3.48	5.89 ± 3.50	0.937
Femoral head	0.96 ± 0.06	0.96 ± 0.06	0.990	4.59 ± 3.85	4.77 ± 3.01	0.865
Small intestine	0.95 ± 0.05	0.95 ± 0.06	0.463	4.86 ± 2.73	4.52 ± 3.59	0.807

The p-value between the DTH + CPDVH method and the IDVH method was given, and the difference was considered statistically significant if the p-value was less than 0.05.

For rectal cancer, the IDVHs method achieved a mean R^2^ value ≥ 0.95 at the bladder, colon, bilateral femoral head and small intestine with standard deviation ≤ 0.06. The IDVHs method achieved a mean MAE value ≤ 6% at the bladder, colon, bilateral femoral head and small intestine with standard deviations ≤ 4%. No significant differences between the DTH + CPDVH method and IDVHs method in the R^2^ and MAE values were found for the OAR in 15 rectal cancer test cases (p-value ≥ 0.190).

Table 4 illustrates the mean absolute difference between dosimetric parameter achieved by the TPS and dosimetric parameter predicted by the DTH + CPDVH or IDVHs method in 20 nasopharyngeal cancer and 15 rectal cancer test cases. The mean absolute difference between the IDVHs method and the TPS ranged from 1.20 to 2.84 Gy at the maximum dose *D*_*0*_ of the brainstem, spinal cord, chiasm, bilateral lens and bilateral optic nerve for the 20 nasopharyngeal cancer test cases. The mean absolute difference between the IDVHs method and the TPS ranged from 1.6 to 2.64% at *V*_*30*_ and *V*_*40*_ of bilateral parotid and bilateral temporal lobe for the 20 nasopharyngeal cancer test cases.

**Table 4:** Mean absolute difference between the dosimetric parameter achieved by the TPS and the dosimetric parameter predicted by the DTH + CPDVH or IDVHs method.

OAR	Parameters	*Δ*_*DTH*+*CPDVH*_	*Δ*_*IDVHs*_	p-value
**Nasopharyngeal cancer**				
Brainstem	*D*_*0*_ (Gy)	2.48 ± 2.23	2.44 ± 2.28	0.204
Spinal cord	*D*_*0*_ (Gy)	2.96 ± 2.16	2.16 ± 1.74	0.179
Chiasm	*D*_*0*_ (Gy)	2.08 ± 2.52	2.24 ± 3.02	0.324
Lens	*D*_*0*_ (Gy)	1.28 ± 0.84	1.20 ± 1.02	0.763
Optic nerve	*D*_*0*_ (Gy)	2.84 ± 4.47	2.84 ± 4.06	0.458
Parotid	*V*_*40*_ (%)	3.20 ± 4.54	2.64 ± 2.51	0.518
Temporal lobe	*V*_*30*_ (%)	1.80 ± 2.68	1.60 ± 2.99	0.601
**Rectal cancer**				
Bladder	*V*_*40*_ (%)	8.97 ± 7.14	9.54 ± 7.28	0.691
Colon	*V*_*40*_ (%)	2.76 ± 2.48	3.19 ± 2.56	0.814
Femoral head	*V*_*40*_ (%)	0.92 ± 0.71	1.08 ± 1.00	0.733
Small intestine	*V*_*30*_ (%)	4.27 ± 3.35	2.89 ± 3.82	0.087

For the DTH + CPDVH method, *Δ*_*DTH*+*CPDVH*_ =|*P*_*DTH*+*CPDVH*_*-P*_*TPS*_|; for the IDVHs method, *Δ*_*IDVHs*_ =|*P*_*IDVHs*_*-P*_*TPS*_|, where *Δ* represents the absolute difference between the dosimetric parameter achieved by the TPS and the dosimetric parameter predicted by the prediction method in the testing cases. *P* represents the corresponding parameter. The data shown are the means and standard deviations of the respective parameters for the 20 nasopharyngeal cancer and 15 rectal cancer test cases.

For the 15 rectal cancer test cases, the mean absolute difference between the IDVHs method and the TPS at *V*_*30*_ and *V*_*40*_ ranged from 1.08% to 9.54% for all OARs. The mean absolute difference between the IDVHs method and the TPS at *V*_*30*_ and *V*_*40*_ was not more than 3.5% at the colon, bilateral femoral head and small intestine with standard deviations ≤ 4%. As shown in Table 4, no significant differences were found between the DTH + CPDVH method and the IDVHs method for all OARs (p-value ≥ 0.087). Both methods achieved a comparable predicted result.

**Figure 4:**
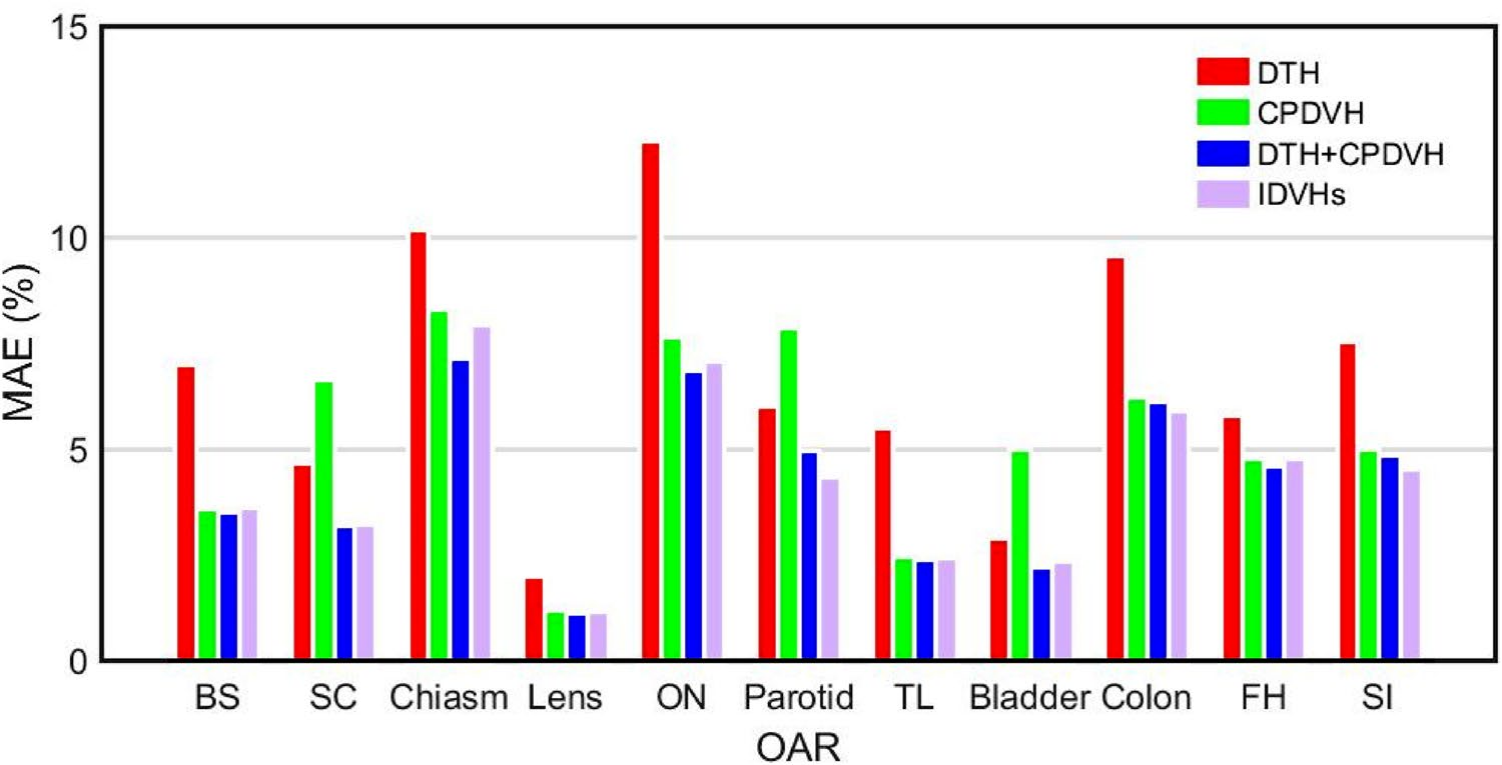
The mean MAE of different prediction methods. *BS* brainstem, *SC* spinal cord, *ON* optic nerve, *TL* temporal lobe, *FH* femoral head, *SI* small intestine.

Figure 4 illustrates the mean MAE value of different DVH prediction methods in the 20 nasopharyngeal cancer and 15 rectal cancer cases. As shown in Fig. 4, both the IDVHs and DTH + CPDVH methods achieved comparable mean MAE value, which was consistent with the results of OAR dosimetric parameters prediction of the two methods.

### PTV DVH prediction accuracy of the IDVHs method

The comparison between the PTV dose coverage generated by the TPS and the PTV dose coverage predicted by the IDVHs method was shown in Table 5. For the PTV70, PTV60 and PTV54 of nasopharyngeal cancer, the mean absolute percentage difference between the plans generated by the TPS vs predicted by the IDVHs method in *D*_*98*_, *D*_*95*_ and *D*_*1*_ ranged from 1.06% to 3.15% in the 20 nasopharyngeal cancer test cases with standard deviations ≤ 3%. For the PTV50 and PTV45 of the rectal cancer cases, the mean absolute percentage difference between the plans generated by the TPS vs predicted by the IDVHs method in *D*_*98*_, *D*_*95*_ and *D*_*1*_ ranged from 0.85% to 3.74% in the 15 rectal cancer test cases with standard deviations ≤ 4%.

**Table 5:** Comparison between the PTV dose coverage achieved by the TPS and the PTV dose coverage predicted by the IDVHs method.

Structures	Parameters	TPS (Mean ± SD)	IDVHs (Mean ± SD)	*diff* (%)	p-value
**Nasopharyngeal cancer**					
PTV70	*D*_*98*_ (Gy)	69.85 ± 1.25	69.56 ± 1.28	2.63 ± 2.08	0.836
	*D*_*95*_ (Gy)	70.76 ± 0.54	70.22 ± 1.95	2.11 ± 1.58	0.307
	*D*_*1*_ (Gy)	75.60 ± 1.00	75.55 ± 0.85	1.06 ± 1.09	0.848
	*UI*	1.07 ± 0.03	1.06 ± 0.02	2.02 ± 1.46	0.296
PTV60	*D*_*98*_ (Gy)	61.33 ± 0.94	61.95 ± 1.32	2.12 ± 1.72	0.052
	*D*_*95*_ (Gy)	62.88 ± 1.07	63.17 ± 1.55	2.57 ± 2.43	0.201
PTV54	*D*_*98*_ (Gy)	53.60 ± 1.62	53.88 ± 1.34	3.15 ± 2.36	0.562
	*D*_*95*_ (Gy)	55.44 ± 1.37	55.65 ± 1.15	2.45 ± 2.21	0.236
**Rectal cancer**					
PTV50	*D*_*98*_ (Gy)	50.83 ± 1.04	50.69 ± 1.74	2.95 ± 2.87	0.286
	*D*_*95*_ (Gy)	51.59 ± 0.94	51.13 ± 1.66	2.57 ± 2.86	0.396
	*D*_*1*_ (Gy)	54.10 ± 0.62	54.12 ± 0.62	0.85 ± 0.76	0.550
	*UI*	1.04 ± 0.02	1.05 ± 0.03	2.63 ± 2.68	0.650
PTV45	*D*_*98*_ (Gy)	46.11 ± 0.74	45.86 ± 0.99	3.74 ± 3.85	0.753
	*D*_*95*_ (Gy)	47.05 ± 0.83	46.85 ± 1.76	3.22 ± 3.63	0.995

The data shown are the means and standard deviations of the respective parameters for the 20 nasopharyngeal cancer and 15 rectal cancer patients. *diff* represents the mean absolute percentage difference between the PTV dose coverage achieved by the TPS and the PTV dose coverage predicted by the IDVHs method. (*diff* =|*P*_*IDVHs*_*-P*_*TPS*_|/*P*_*TPS*_ × *100%*), where, *P* represents the corresponding parameter.

For the PTV70 in the nasopharyngeal cancer cases, the TPS and IDVHs method achieved *UI* values of 1.07 ± 0.03 and 1.06 ± 0.02, respectively. For the PTV50 in the rectal cancer cases, the TPS and IDVHs method achieved *UI* values of 1.04 ± 0.02 and 1.05 ± 0.03, respectively. No significant difference was found in the *UI* values between the TPS and IDVHs method. Likewise, no significant difference was found in PTV dose coverage at *D*_*98*_, *D*_*95*_ and *D*_*1*_ between the TPS and IDVHs method for both nasopharyngeal cancer and rectal cancer cases.

Supplemental Fig. 1 shows the comparison between the predicted PTV DVH using the IDVHs method versus the TPS. Two PTVs, PTV70 of the nasopharyngeal cancer cases and PTV50 of the rectal cancer cases, are shown in detail. As shown in Supplemental Fig. 1, for the nasopharyngeal cancer, the predicted DVHs of PTV70 are close to the DVHs achieved by the TPS in 18/20 test cases, except for cases #1 and #10. For the rectal cancer, except for cases #1, #8 and #12, most predicted DVHs of PTV50 are close to the DVHs achieved by the TPS in the 15 test cases.

Supplemental Fig. 2 illustrates the R^2^ value of different PTVs of all the testing cases. Regarding the R^2^ value, 17/20 cases in PTV70, 20/20 cases in PTV60, 11/20 cases in PTV54, 11/15 cases in PTV50 and 13/15 cases in PTV45 are located at within 0.9 ~ 1. The IDVHs method achieved a mean R^2^ value at PTV70, PTV60, PTV54, PTV50 and PTV45 of 0.95, 0.98, 0.93, 0.92 and 0.95, respectively.

## Discussion

The difference between the method using only the DTH and the IDVHs method or the DTH + CPDVH method at the mean MAE value is rather large (see Fig. 4). The use of a set of individual DVHs (IDVHs) improves the prediction accuracy of the DVHs of OARs that are partially surrounded or overlapped by PTV (such as spinal cord, parotid and bladder), and they cannot be predicted accurately employing the superimposed dosimetric information (such as the CPDVH). The results show that the better OAR DVH prediction using a set of individual DVHs containing the direction-dependent dosimetric information.

The patient geometric information-based three-dimensional (3D) dose prediction model has been widely studied and reported in recent years and can provide the predicted dosimetric results for OAR and PTV^6–9^. Fan et al. applied a deep learning-based model to predict the 3D dose distribution for head-and-neck cancer and no significant difference was found between the predicted plan (Pred) and the manually optimized plan (MO) at *D*_*95*_ of PTV_60_ (mean, 59.4 Gy vs 59.6 Gy, p = 0.05) and PTV_54_ (mean, 53.9 Gy vs 53.7 Gy, p = 0.28)^8^. For comparison, the difference between the predicted plan and the manually optimized plan at *D*_*95*_ of different PTVs was calculated as follows: *ε* =|*Pred*-*MO*|/*MO* × *100%*, where *Pred* represented the average *D*_*95*_ of PTVs in the predicted plans; *MO* represented the average *D*_*95*_ of PTVs in the manually optimized plans*.* Compared with their results^8^, the difference between the 3D dose prediction method and the IDVHs method at *D*_*95*_ of different PTVs was (PTV_60_, *ε*, 0.34% vs 0.46%) and (PTV_54_, *ε*, 0.37% vs 0.38%). Recently, Song et al. proposed a deep neural network to predict the 3D dose of rectal cancer patients, and the results showed that no significant difference between the DeepLabv3 + prediction results (Dose_DeepLabv3+_) and the Dose_approved_ was found at *D*_*98*_ of PTV_50_ (mean, 49.19 Gy vs 49.07 Gy, p = 0.23)^10^. Compared with their prediction results^9^, the difference between the deep neural network method and the IDVHs method at *D*_*98*_ of PTV_50_ was (*ε*, 0.24% vs 0.28%). The predicted PTV dosimetric results of this study are comparable to the results of Fan et al.^8^ and Song et al.^9^.

Considering that the geometry differences for distinct diseases may affect the DVH prediction accuracy of the IDVHs method, in this work, DVH prediction was studied for nasopharyngeal carcinoma of the head-and-neck and rectal cancer of the abdomen. The results showed the feasibility and effectiveness of predicting the OAR and PTV DVH in these two diseases using the IDVHs method. Future studies on the predictive value of the IDVHs method in other diseases are warranted.

For the IMRT plan with a fixed irradiation angle, the irradiation field arrangement could be used to establish the corresponding conformal plan to extract the features of the OAR and target. For other diseases using a 5-fields or 7-fields arrangement, the corresponding field arrangement of the conformal plan can be adjusted accordingly. The VMAT plan, which samples many irradiation fields to calculate the dosimetric information, is time consuming. Perhaps, sampling less equally spaced irradiation fields can achieve similar prediction accuracy with 9 equally spaced fields. The difference in prediction accuracy when sampling different equally spaced irradiation fields will be studied in the future.

## Conclusion

This work evaluated the performance of the IDVHs method in predicting both the OAR and PTV DVH for IMRT and VMAT plans. The results indicated that the IDVHs method is comparable to the DTH + CPDVH method in accurately predicting the OAR DVH. The IDVHs method quantified the input features of the PTV and showed reliable PTV DVH prediction. Therefore, the IDVHs method can provide more comprehensive guidance information for radiotherapy treatment plan quality evaluation and plan generation.

## Supplementary Information

Supplementary Information

## Data Availability

The datasets generated and/or analysed during the current study are available from the corresponding author on request.
