# Supplementary material for: Evaluation of dose-volume histogram prediction for organ-at risk and planning target volume based on machine learning
Source: Sci Rep. 2021 Feb 4;11:3117. doi: 10.1038/s41598-021-82749-5 (PMC7862493; doi:10.1038/s41598-021-82749-5)
Supplement: Supplementary file 1 — Supplementary Information [file 41598_2021_82749_MOESM1_ESM.docx]

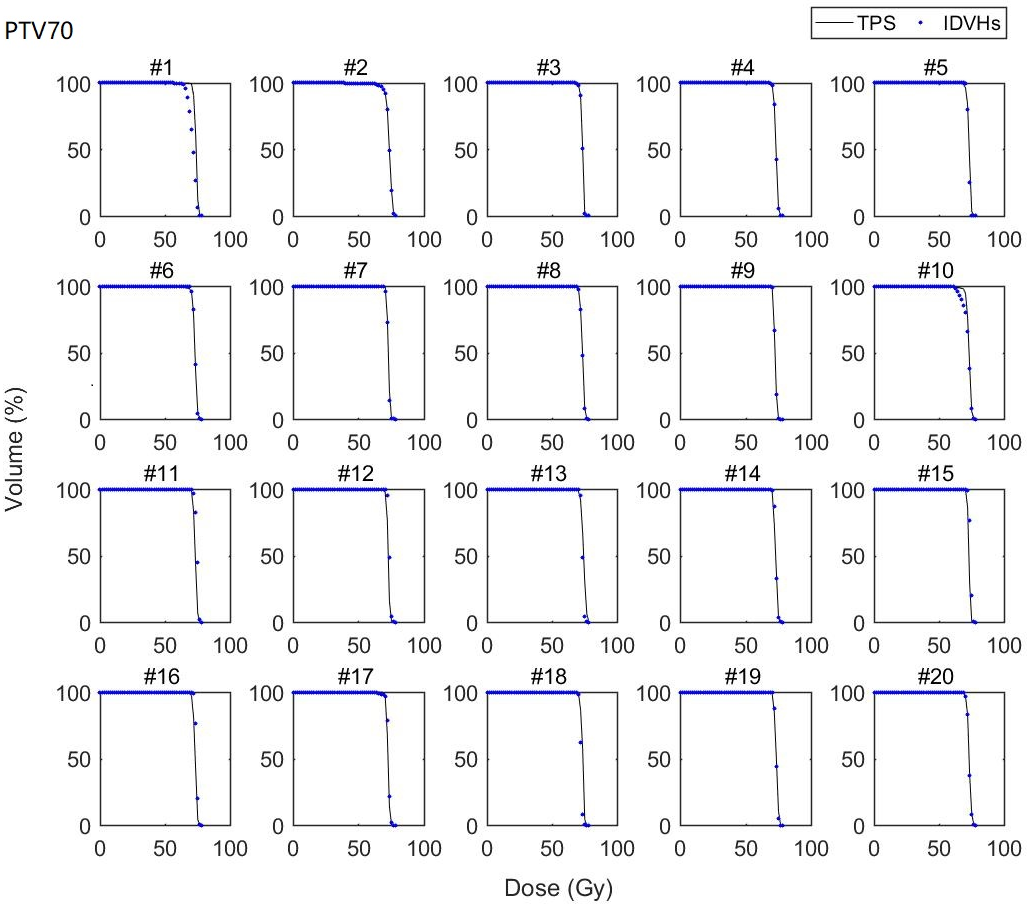


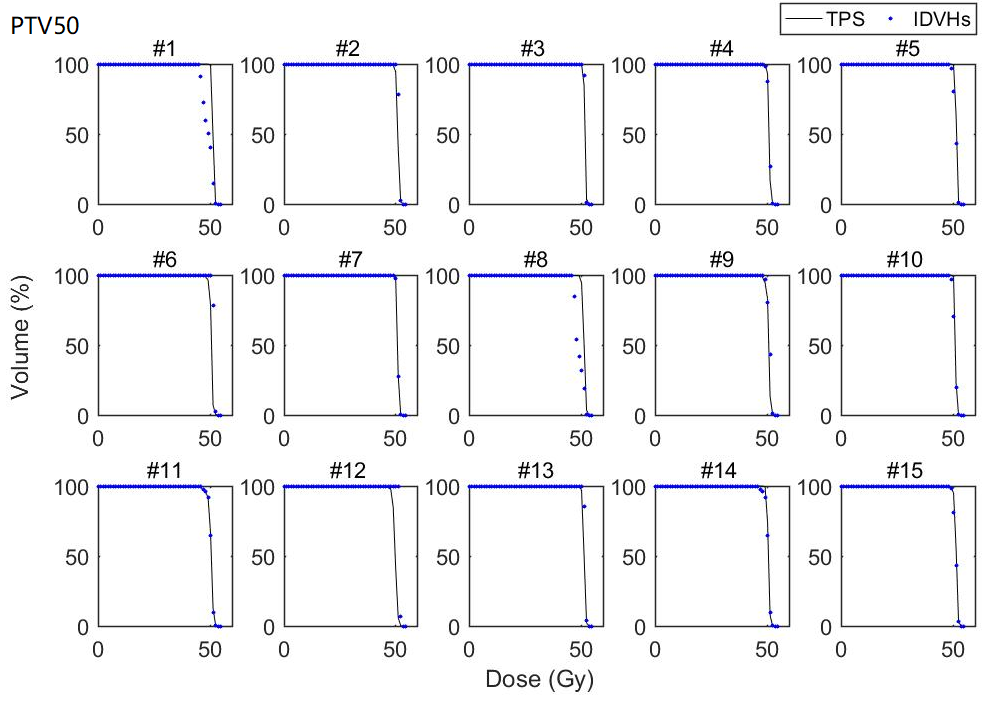


Supplemental Figure 1. Comparison between the PTVs DVHs predicted by the IDVHs method (blue scatter) and the PTVs DVHs achieved by the TPS (black solid line). The PTV70 of nasopharyngeal cancer and the PTV50 of rectal cancer are displayed in detail.


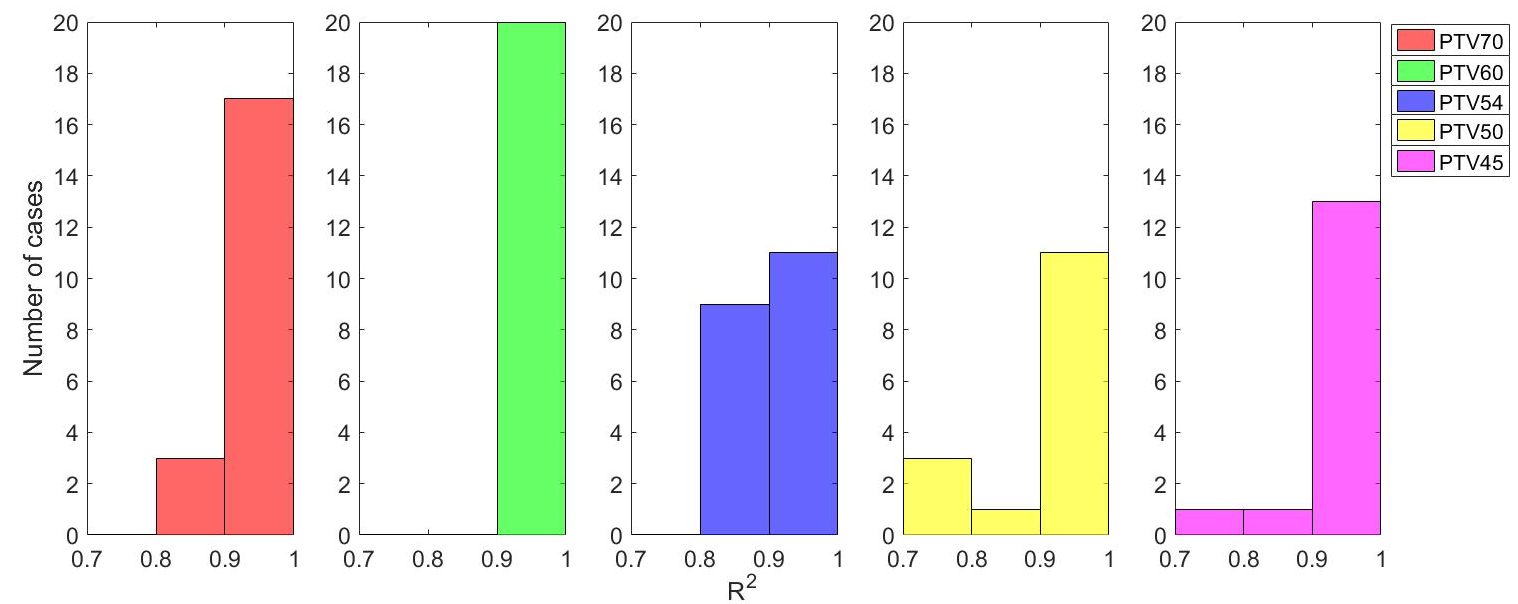


Supplemental Figure 2. PTV R^2^ values for the IDVHs prediction method.
